# Commentary: Understanding emerging psychopathology through symptom networks—A commentary on Häfeli et al. (2026)

**DOI:** 10.1002/jcv2.70165

**Published:** 2026-08-21

**Authors:** Antonia N. Kaczkurkin

**Affiliations:** ^1^ Department of Psychology Vanderbilt University Nashville Tennessee USA

**Keywords:** adolescence, developmental psychopathology, network analysis, prevention, transdiagnostic

The study by Häfeli et al. (2026) published in the 2026 Special Issue of *JCPP Advances* represents an important step in the evolution of developmental psychopathology. Rather than asking which disorders predict later disorders, or even which symptom domains precede others, the authors examine how individual symptoms interact over time across multiple forms of emerging psychopathology in adolescents. Their findings that uncontrollable worry, body dissatisfaction, and weight concerns occupy influential positions in longitudinal symptom networks, while irritability and low self‐esteem bridge multiple diagnostic domains offer more than another demonstration of transdiagnostic overlap. They invite us to reconsider what the earliest manifestations of psychopathology actually represent and, perhaps more importantly, how the field should conceptualize prevention.

The most interesting aspect of this work is not that certain symptoms are central. It is that the most influential symptoms are not obviously tied to any single diagnostic category. Worry is often considered an anxiety symptom, body dissatisfaction an eating disorder symptom, and low self‐esteem a depressive symptom. Yet in this sample, these experiences appear less like markers of individual disorders than organizing principles around which multiple forms of psychopathology begin to cluster. This distinction has important implications for how developmental psychopathology conceptualizes risk.

For decades, the field of developmental psychopathology has emphasized equifinality and multifinality: different developmental pathways can culminate in similar disorders, while similar early experiences can lead to different outcomes (Valentino & Edler, 2024). Network approaches provide an appealing framework because they operationalize these principles at the level of symptoms rather than diagnoses. Häfeli and colleagues extend this perspective by examining symptom interactions in adolescents before the emergence of diagnosable disorders, providing a window into the earliest stages of psychopathology. Their findings therefore contribute to an increasingly important shift in the field, from studying disorders after they emerge to studying the dynamic processes that precede them.

Yet these findings also raise a broader question: what exactly does it mean for a symptom to be “central”? Network models often encourage the interpretation that central symptoms are causal drivers, implying that intervening on these symptoms should produce cascading improvements throughout the system (Borsboom, 2017). Causality is an intuitively attractive idea, particularly for prevention. However, centrality should perhaps be viewed less as evidence of causality than as evidence of developmental importance. Highly connected symptoms themselves may not drive the emergence of psychopathology. Instead, they may represent points of developmental convergence through which multiple biological, cognitive, and environmental influences become behaviorally expressed.

This distinction matters. If worry predicts later depressive symptoms, as Häfeli and colleagues report, does reducing worry necessarily prevent depression? Possibly. However, evidence that network centrality identifies particularly consequential intervention targets remains limited. In one direct test, changes in more central symptoms were more strongly associated with changes elsewhere in the same symptom network, but this pattern did not generalize beyond the measure used to construct the network, and how frequently a symptom was endorsed was a more consistent predictor of which symptoms were influential (Rodebaugh et al., 2018). More broadly, worry may serve as an observable manifestation of underlying vulnerabilities in emotion regulation, uncertainty tolerance, or cognitive control. Similarly, body dissatisfaction may not itself generate psychopathology so much as reflect adolescents' increasing sensitivity to social evaluation, identity formation, and cultural appearance ideals. These symptoms may therefore function less as independent causal agents than as developmental “hubs” where multiple risk processes become visible.

A developmental hub interpretation provides a way to integrate network approaches with established developmental science. Network models can identify where risk becomes visible and interconnected at the symptom level, while developmental frameworks can help explain why certain symptoms become influential at specific points in development and under particular environmental conditions. A symptom's centrality may therefore be meaningful not because it reveals a universal driver of psychopathology, but because it identifies a process that has particular significance within a given developmental context. The next step is to understand what gives these symptoms their influence and whether the same symptoms remain influential across developmental periods, individuals, and environments.

The prominence of worry in this study is particularly noteworthy. Across contemporary developmental models, repetitive negative thinking, a combination of worry and rumination, has emerged as a remarkably consistent transdiagnostic process. Worry and rumination have been implicated in anxiety, depression, psychosis, and self‐harm in young people, and interventions designed specifically to target repetitive negative thinking reduce anxiety and depressive symptoms to a comparable degree, largely in subthreshold, prevention‐focused samples aged 14 and older (Egan et al., 2025). Häfeli and colleagues strengthen this literature by showing that uncontrollable worry is not merely associated with multiple symptoms cross‐sectionally but exerts the strongest predictive influence of any node in the temporal network, forecasting all other anxiety symptoms as well as depressed mood, low self‐esteem, and sleep problems.

Perhaps more surprising is the equally prominent role of body dissatisfaction and weight concerns. Traditionally, body image concerns have been viewed primarily through the lens of eating disorders. The present findings suggest a considerably broader role. In the temporal network, body dissatisfaction predicted depressive symptoms, psychotic‐like experiences, and bipolar symptoms, while closely related weight concerns predicted anxiety, depressive, and obsessive‐compulsive symptoms. Rather than viewing negative body image as disorder‐specific, these findings support an emerging perspective that body image represents a core developmental challenge of adolescence itself, consistent with prospective evidence that body dissatisfaction predicts the onset of depression in adolescent girls and boys (Bornioli et al., 2021).

The findings of Häfeli and colleagues raise intriguing questions about why body dissatisfaction is so influential during this developmental period. Adolescence involves profound changes in physical appearance, peer relationships, identity formation, and social comparison. Body dissatisfaction may therefore reflect more than appearance concerns alone; it may index broader concerns about self‐worth, social belonging, and perceived acceptance. If so, interventions that focus narrowly on eating pathology may underestimate the broader developmental significance of body image.

This leads to a larger issue for prevention science. Historically, preventive interventions have largely mirrored diagnostic categories: anxiety prevention, depression prevention, eating disorder prevention, and so forth. Yet if symptoms rather than disorders represent the earliest manifestations of psychopathology, prevention may need to become organized around developmental processes rather than diagnostic outcomes. One can imagine school‐based programs designed around reducing repetitive negative thinking, improving body image flexibility, strengthening emotion regulation, or enhancing self‐concept, with the expectation that such interventions would influence multiple downstream outcomes simultaneously. Such approaches would align well with recent calls for transdiagnostic prevention (Shah et al., 2020) while also acknowledging that adolescents rarely fall into clearly distinct diagnostic categories.

However, translating symptom networks into preventive interventions will require answering a more difficult question than identifying central nodes. We need to understand whether central symptoms are malleable. Some highly connected symptoms may prove surprisingly resistant to change because they reflect broader developmental contexts. Body dissatisfaction, for example, is shaped not only by individual cognition but by peer cultures, family attitudes, media exposure, and increasingly pervasive digital environments. Similarly, worry may arise partly from normative developmental changes in abstract thinking and future‐oriented cognition. If central symptoms are embedded within larger ecological systems, effective intervention may require modifying environments as much as individuals.

This observation highlights one limitation of symptom‐only approaches more generally. Symptoms do not exist in isolation from contexts. Development unfolds within families, schools, peer groups, digital environments, and broader sociocultural systems. Networks composed exclusively of psychological symptoms necessarily omit many of the processes that generate and sustain those symptoms.

The next generation of developmental network research may therefore benefit from moving beyond symptom networks toward models that situate symptoms within broader developmental systems. Rather than including only internal psychological experiences, future models might integrate peer victimization, sleep patterns, social media use, family conflict, school connectedness, pubertal development, inflammatory markers, or passive digital sensing data. Such networks would more closely reflect the ecological reality in which adolescent psychopathology develops. Indeed, one could argue that the most exciting future direction is not building larger symptom networks but building richer developmental networks that span biological, psychological, social, and environmental levels of analysis. This would bring network models into closer dialog with developmental systems theory rather than positioning them as alternatives to existing frameworks.

Another important implication concerns developmental timing. Häfeli and colleagues examine adolescents spanning approximately ages 11–17, a developmental period that encompasses the onset of many psychiatric disorders. Yet adolescence is not a single developmental stage. Early adolescence differs substantially from middle and late adolescence in pubertal maturation, social cognition, autonomy, peer relationships, and identity development. The symptoms that occupy central positions at age 12 may differ substantially from those that become influential at age 17. Future work may therefore shift from estimating static developmental networks toward estimating developmental trajectories of networks themselves. Rather than asking which symptoms are central, we may ask how centrality changes across puberty, across transitions, or following major developmental milestones.

Relatedly, the present findings remind us that psychopathology is dynamic not simply because symptoms predict one another but because adolescents themselves are changing rapidly. Development introduces new vulnerabilities, new protective factors, and new environmental exposures. Symptom networks therefore exist within a moving developmental landscape.

The study also illustrates both the promise and the limitations of more sophisticated statistical methods. Panel graphical vector autoregression represents a considerable methodological advance over cross‐sectional networks by separating contemporaneous and temporal associations. Nevertheless, developmental psychopathology should resist the temptation to equate increasingly complex models with increasingly complete explanations (Bringmann et al., 2022). Sophisticated statistical models identify patterns in data; developmental theory explains why those patterns exist. The most productive future research will likely combine methodological innovation with equally ambitious theoretical development. Network science needs developmental theory just as much as developmental theory can benefit from network science.

Perhaps the most significant contribution of Häfeli and colleagues is therefore conceptual rather than statistical. Their work shifts attention away from disorders and toward the earliest observable building blocks of psychopathology. This perspective aligns closely with broader movements across psychiatry emphasizing dimensionality, clinical staging, and transdiagnostic mechanisms (Shah et al., 2020). It suggests that prevention may be most effective when focused on common developmental processes before disorder‐specific symptom constellations have fully crystallized. Ultimately, however, the goal is not to identify increasingly influential symptoms but to explain their influence. Network models can identify where developmental systems become vulnerable, but developmental science remains essential for explaining how those vulnerabilities emerge.

## CONCLUSION

Viewed in this light, the findings reported by Häfeli and colleagues represent less a conclusion than an invitation. Their work encourages the field to move beyond diagnostic categories, beyond cross‐sectional associations, and perhaps even beyond symptom networks themselves. The next frontier will be integrating network approaches with developmental systems perspectives that encompass biology, cognition, relationships, and environments across time. If successful, this integration could transform prevention science, from attempting to prevent disorders after developmental pathways are already established to modifying the processes through which psychopathology first emerges.

That may ultimately be the most important implication of this study. The earliest signs of mental illness may not simply be milder forms of adult disorders. Instead, they may be dynamic developmental processes that only later differentiate into recognizable diagnostic syndromes. Understanding those processes, not merely mapping their connections, may represent the next major challenge for developmental psychopathology.

## AUTHOR CONTRIBUTIONS

**Antonia N. Kaczkurkin**: Conceptualization; writing—original draft; writing—review and editing.

## CONFLICT OF INTEREST STATEMENT

The author declares no conflicts of interest.

## ETHICAL CONSIDERATIONS

Ethics requirements do not apply to this article, as it is a commentary and involved no human participants or new data collection.

## AI STATEMENT

The authors acknowledge the use of ChatGPT (GPT‐5.6 Sol; OpenAI, 2026) for language polishing, copy‐editing, and improving the readability and clarity of the commentary. The AI was used solely for editorial assistance and did not generate the substantive ideas, arguments, or conclusions presented in the commentary. All AI‐assisted edits were reviewed, revised as appropriate, and verified by the author, who takes full responsibility for the final content.

## Data Availability

Data sharing is not applicable to this article as no new data were collected or analyzed for this commentary article.
